# Surgical management of an acquired aortic interruption suspected from severe aortic coarctation in an adult with massive collaterals

**DOI:** 10.1186/s13019-026-04600-0

**Published:** 2026-07-17

**Authors:** Min Qiu, Chengyi Hui, Qikun Zhu, Zewen Chen, Yong Zhang, Shusheng Wen, Hujun Cui

**Affiliations:** 1 Department of Cardiovascular Surgery, Guangdong Provincial People’s Hospital (Guangdong Academy of Medical Sciences), Guangdong Cardiovascular Institute, Southern Medical University, Guangzhou, 510080 China; 2 Guangdong Provincial Key Laboratory of South China Structural Heart Disease, Guangdong Provincial People’s Hospital (Guangdong Academy of Medical Sciences), Guangdong Cardiovascular Institute, Southern Medical University, 106 Zhongshan Er Rd, Guangzhou, 510080 China

**Keywords:** Acquired interruption, Collateral circulation, Extra-anatomic bypass

## Abstract

**Background:**

Interrupted aortic arch (IAA) is a severe congenital anomaly typically fatal in infancy without surgical intervention. Adult presentation of isolated IAA is exceedingly rare and usually relies on an extensive compensatory collateral vascular network.

**Case presentation:**

We present the case of a 25-year-old man who presented with malignant hypertension. Computed tomography angiography with 3D reconstruction revealed complete occlusion of the aortic arch distal to the left subclavian artery, accompanied by a massive collateral circulation derived from dilated intercostal and internal mammary arteries. Open surgical repair was performed via a left posterolateral thoracotomy. Intraoperative findings identified an atretic fibrous cord at the occlusion site, suggesting an extreme progression of severe coarctation of the aorta into an acquired interruption. An 18-mm Dacron tube graft was successfully interposed between the arch and descending aorta. Crucially, this was performed as an off-pump extra-anatomic bypass without cardiopulmonary bypass. The postoperative course was uneventful. At 4 months, his blood pressure was significantly improved, though a small residual gradient persisted.

**Conclusion:**

This case suggests that severe coarctation can progress to an acquired atresia mimicking IAA. Off-pump extra-anatomic bypass is a safe and effective strategy expected to alleviate refractory hypertension and prevent long-term cardiovascular and cerebrovascular complications in heavily collateralized adults.

## Background

Interrupted aortic arch (IAA) and coarctation of the aorta (CoA) are severe congenital anomalies associated with high mortality if left untreated [1]. Without surgical intervention, 50% of patients with CoA succumb by the age of 10 years, and up to 90% of those with IAA die within the first year of life [2]. While isolated IAA or severe CoA is exceedingly rare in adults, it can manifest in patients who have developed an extensive compensatory collateral circulation [2]. Pathophysiologically, IAA is often considered the most extreme spectrum of CoA [3]. Furthermore, progressive luminal occlusion in severe CoA can occasionally culminate in an acquired complete interruption (sometimes referred to as acquired atresia), clinically and anatomically mimicking a primary congenital IAA [4]. However, literature emphasizing the progression of the CoA to a complete interruption in adult or adolescent remains scarce. In this case report, we describe the diagnosis and successful off-pump extra-anatomic surgical management of an adult patient with isolated acquired aortic interruption accompanied by an extensive collateral circulation.

## Case presentation

A 25-year-old man was referred to our institution for the management of an aortic arch anomaly. He initially presented to a community hospital two years prior with headaches secondary to malignant hypertension, recording a peak blood pressure (BP) exceeding 180/100 mmHg. Accordingly, antihypertensive therapy was initiated with nifedipine sustained-release tablets at a dosage of 30 mg once daily, and the patient was counseled to undergo further evaluation at a tertiary care facility. The patient did not comply with this medical advice. Over the subsequent 2 years, blood pressure control remained suboptimal, with fluctuations around 140/90 mmHg. One month prior to admission, a complete aortic computed tomography angiography (CTA) performed locally demonstrated coarctation of the aortic arch with focal luminal occlusion and the development of collateral circulation. On the basis of this radiological finding, the patient was referred to our institution for definitive management. The patient and his families denied any discomfort other than headache during childhood and throughout the disease process. He had normal cognitive and physical development without dysmorphic features suggestive of genetic syndromes.

**Fig. 1:**
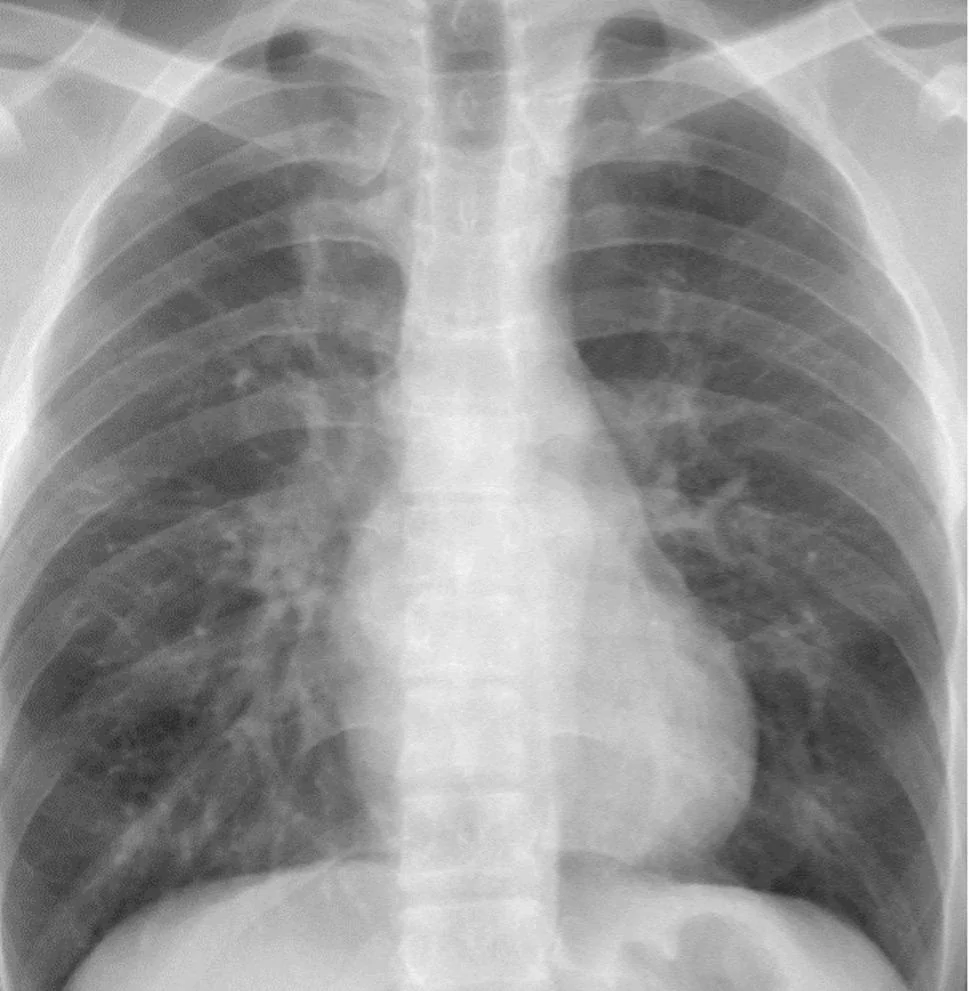
Preoperative chest radiograph

The image demonstrates a concavity at the aortic knob with a normal cardiac silhouette and mild rib notching typical of collateral engorgement.

Physical examination revealed a distinct radio-femoral pulse delay. Blood pressure measurements of the four extremities yielded the following results: left upper extremity, 141/93 mmHg; left lower extremity, 105/70 mmHg; right upper extremity, 139/93 mmHg; right lower extremity, 110/81 mmHg. Laboratory test and electrocardiogram showed no special abnormal results. Chest radiograph revealed a concavity at the aortic knob with a normal cardiac silhouette and mild bilateral rib notching typical of chronic intercostal collateral engorgement (Fig. 1).

Preoperative transthoracic echocardiography showed aortic sinus dilatation (38 mm) and mid-distal descending aortic stenosis. The aortic valve was morphologically normal and tricuspid without left ventricular outflow tract (LVOT) obstruction. Reflecting the chronic cardiac impact of severe afterload, secondary left ventricular hypertrophy was noted with a left ventricular mass index of 132.64 g/m² (Left Ventricular End-Diastolic Dimension (LVDd: 43 mm), Interventricular Septal End-Diastolic Thickness (IVST: 12 mm), Left Ventricular Posterior Wall Thickness (LVPWT: 12 mm)), and a preserved ejection fraction of 69%. Mild aortic regurgitation was present. Cardiac catheterization for Left Ventricular End-Diastolic Pressure (LVEDP) and selective angiography was not performed. The high-resolution 3D CTA and echocardiography provided sufficient anatomical mapping for our planned extra-anatomic approach, and we opted to avoid the procedural stroke risks associated with catheterizing a severely hypertensive patient with profound vascular tortuosity.

**Fig. 2:**
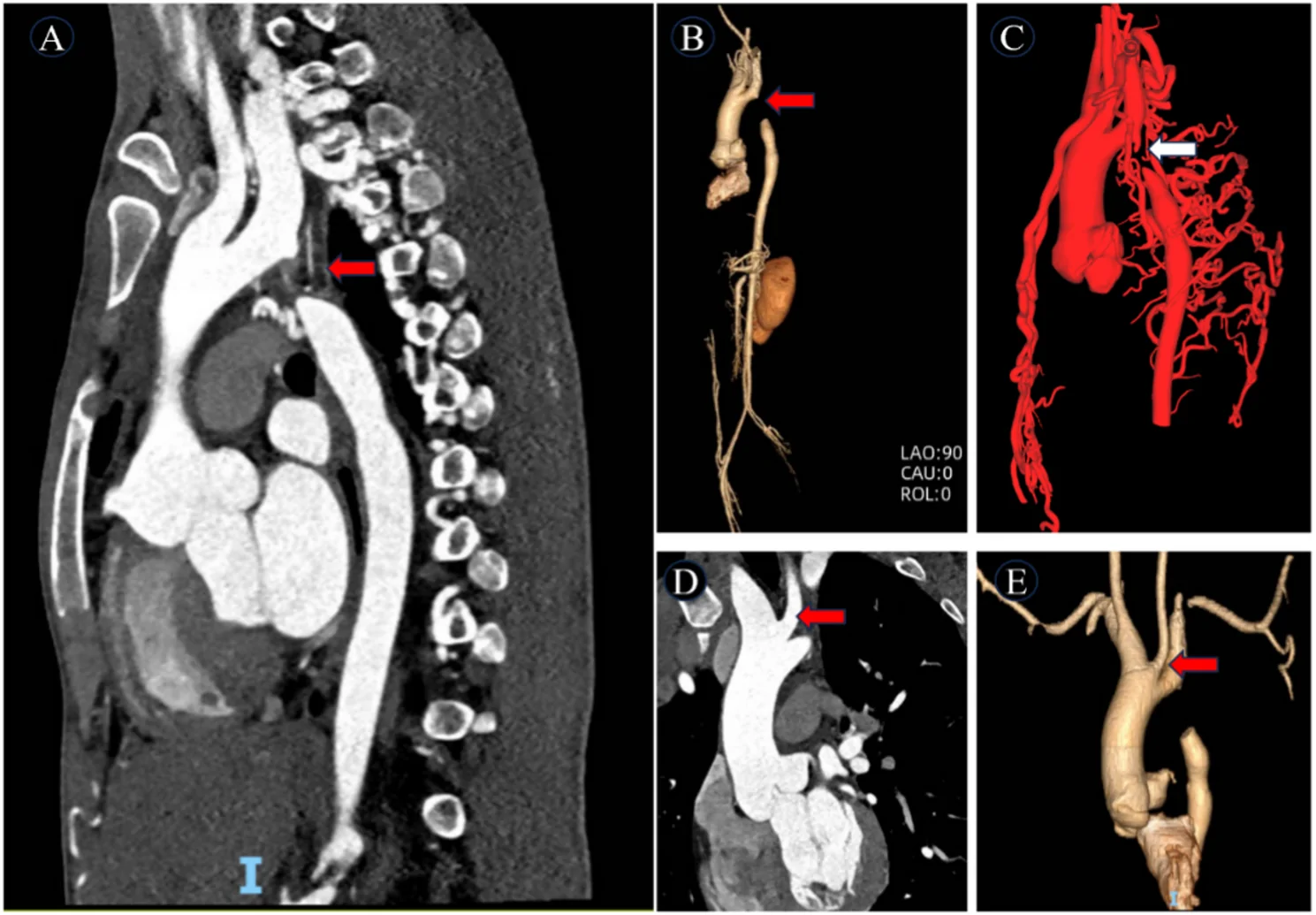
Preoperative contrast-enhanced cardiac CT and Cardiac 3D reconstructions. The imaging demonstrates focal discontinuity of the aortic arch with extensive surrounding collateral vessels arising from the bilateral subclavian arteries and coursing through the anterior chest wall to the abdominal cavity. (**A**) Two-dimensional CT slices in the left sagittal planes. (**B**) Multiplanar reconstruction images in the left sagittal planes. (**C**) Volume-rendered 3D reconstructions in the left sagittal planes revealing the massive collateral network. Arrows in all panels indicate the site of complete aortic interruption. (**D**,** E**) The adjusted left sagittal planes image shows the brachiocephalic trunk and left common carotid artery originated as a common trunk from the aortic arch (bovine arch configuration). Red arrow shows the trunk

Contrast-enhanced cardiac CT, utilizing multiplanar reconstruction (MPR) and volume-rendered 3D imaging, delineated the precise anatomy: aortic sinus dilatation with an internal diameter of approximately 40 mm; focal discontinuity of the aortic arch (Fig. 2A and B). Furthermore, the 3D volume-rendered models provided a detailed visualization of a massive surrounding collateral network (Fig. 2C). Collateral branches arising from the bilateral subclavian arteries coursed through the parasternal region of the anterior chest wall to the abdominal cavity (Fig. 2C). The bilateral internal mammary arteries and multiple bilateral intercostal arteries were tortuous and dilated, with prominent tortuosity and dilatation of collateral vessels observed below the bilateral diaphragms. A bovine arch configuration was also noted. (Figure 2D and E).

**Fig. 3:**
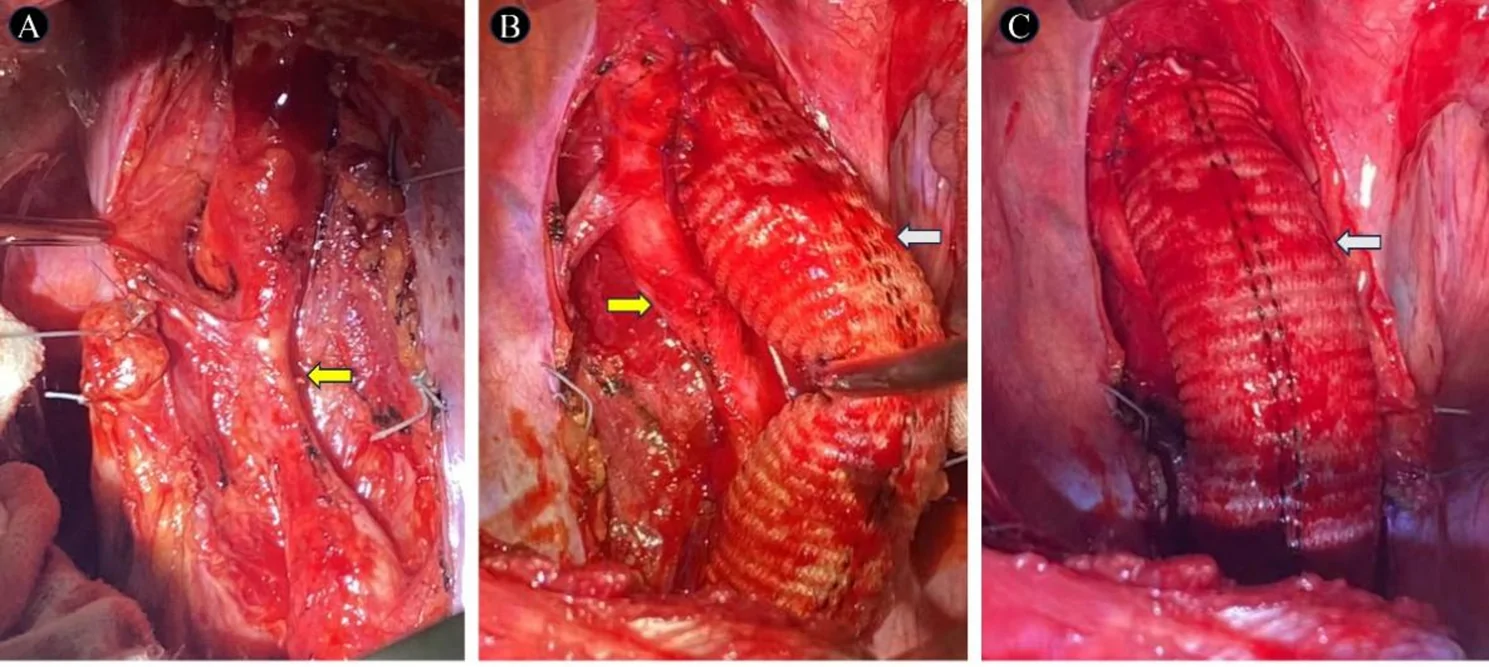
Intraoperative photographs via a left posterolateral thoracotomy. (**A**) Before graft implantation, an atretic fibrous cord (yellow arrow) is seen connecting the interrupted aortic arch to the descending aorta. (**B**, **C**) After implantation, showing the preservation of the atretic fibrous cord (yellow arrows) and the interposed 18-mm Dacron tube graft (red arrows) connecting the arch to the descending aorta

An anatomic repair via sternotomy was considered; however, it requires extensive mobilization of the hypoplastic arch and descending aorta, which is highly morbid in a heavily collateralized adult. Based on our institutional experience and preference for a less invasive alternative, an off-pump extra-anatomic bypass via a left posterolateral thoracotomy was chosen. Intraoperative exploration revealed massive dilation of the intercostal arteries. Because encountering the extensive collateral circulation during thoracic entry and exposure was inevitable, the dissection was performed with the utmost meticulous care, and any interfering engorged collateral vessels were precisely ligated to minimize blood loss. The arch was hypoplastic, sequentially giving rise to the brachiocephalic trunk, left common carotid artery, and left subclavian artery. A 3-cm occlusion of the aortic lumen was identified distal to the origin of the left subclavian artery, connected to the descending aorta only by an atretic fibrous cord (Fig. 3A).

The preoperative invasive arterial systolic pressure difference between right upper and lower extremities was approximately 60mmHg. During the procedure, Cooley clamps were meticulously applied to cross-clamp the distal aortic arch (distal to the left subclavian artery) and the proximal descending aorta. The cross-clamp time was about 25 min. To ensure adequate distal perfusion and prevent paraplegia without cardiopulmonary bypass, simultaneous invasive arterial pressures were continuously monitored. The anesthesia team maintained the lower extremity distal mean arterial pressure (MAP) of > 50–60 mmHg specifically to prevent paraplegia. Because cerebral perfusion remained uninterrupted based on the clamp position, and the robust collateral network naturally maintained adequate distal spinal cord MAP, adjunctive monitoring (such as cerebral/somatic NIRS) and spinal cord protection measures (e.g., cerebrospinal fluid drainage) were deemed unnecessary.

The atretic fibrous cord was intentionally preserved. An 18-mm diameter Dacron tube graft was interposed via end-to-side anastomoses (Fig. 3B-C). Estimated blood loss was 100 mL, and 4 units of blood were transfused. Immediately upon removing the Cooley clamps and restoring graft perfusion, the invasive upper-to-lower limb systolic pressure gradient successfully decreased to approximately 15 mmHg. The patient was discharged on postoperative day 4. The 4-extremity BP disparities were largely resolved (left upper extremity, 109/69 mmHg; left lower extremity, 117/72 mmHg; right upper extremity, 118/75 mmHg; right lower extremity, 117/75 mmHg).

**Fig. 4:**
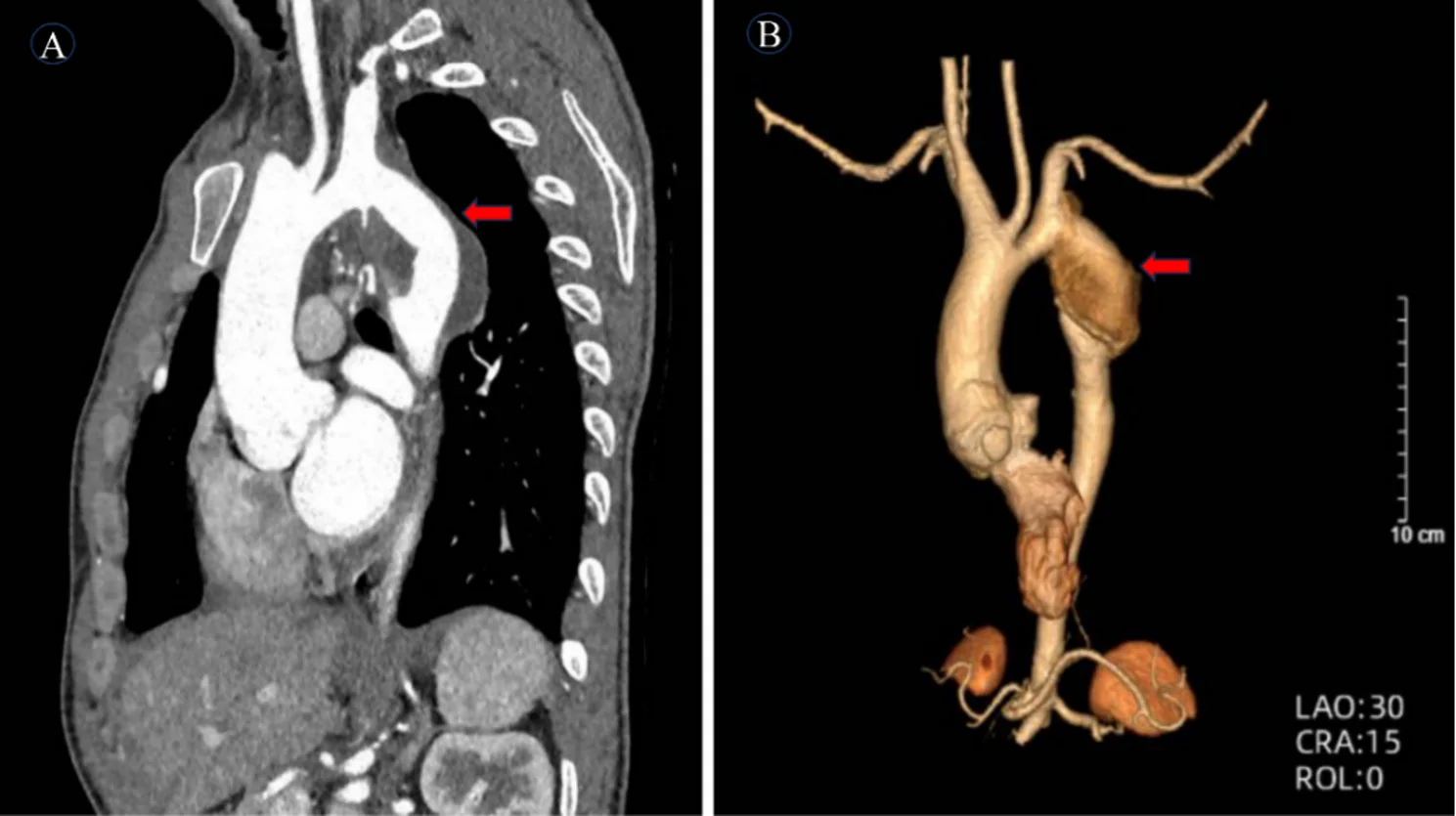
Follow-up cardiac CTA at 4 months postoperatively. The imaging demonstrates a widely patent Dacron graft without evidence of stenosis in the coronal (**A**) and left sagittal (**B**) planes. Arrows indicate the interposed graft

Four months postoperatively, follow-up CTA confirmed a patent graft without stenosis (Fig. 4), and his BP had normalized (117/80 mmHg) on a regimen of metoprolol and fosinopril. Postoperative echocardiography demonstrated stable LV function. Clinically, his previous unprovoked headaches had completely resolved. Furthermore, although the patient did not subjectively perceive diminished exercise capacity prior to surgery due to long-term adaptation, he noted a distinct self-perceived improvement in his overall exercise tolerance compared to his preoperative baseline.

## Discussion

The central anatomical finding in this case—an atretic fibrous cord connecting the proximal arch to the descending aorta—strongly suggests an acquired aortic interruption. Morphologically, a true primary congenital IAA typically presents as a complete anatomic gap. In contrast, acquired interruption represents the extreme end-stage progression of a severe coarctation of the aorta (CoA) culminating in acquired luminal atresia [4]. Without longitudinal imaging from childhood, “progression” remains a hypothesis; however, framing this lesion as an acquired interruption highlights the remarkable capacity of the human vasculature to adapt over decades.

Long-term survival in adult unoperated arch obstructions is exceptionally rare. Survival fundamentally relies on two adaptations: profound myocardial hypertrophy to overcome afterload, and the establishment of a wide, hemodynamically effective collateral network (predominantly the subclavian-intercostal dominant system) acting as a natural bypass [5]. However, conservative management carries the impending risk of cerebrovascular catastrophe driven by medically refractory malignant hypertension, alongside the risk of aneurysm formation or dissection within fragile collaterals [1].

Surgical management of heavily collateralized adult acquired IAA is technically demanding. While several reports, including a similar case have demonstrated the feasibility of off-pump extra-anatomic bypass, the choice of approach requires careful consideration [6, 7]. We opted for a left subclavian-to-descending aortic bypass via left thoracotomy as a less invasive alternative to anatomic repair. This approach relies on institutional experience to minimize surgical trauma, avoids the deep posterior mediastinal dissection required for sternotomy, and circumvents the need for cardiopulmonary bypass [6, 7].

Following the bypass, the patient exhibited significant clinical improvement, though a residual systolic gradient of < 15 mmHg persisted. This gradient is likely multifactorial. Considering the patient’s extremely small body surface area (BSA = 1.40 m²; height 161 cm, weight 44 kg), the chosen 18-mm Dacron graft is physiologically perfectly matched to his systemic requirements (Z-score ≈ + 1.0) according to established normative nomograms for descending aortic dimensions. Therefore, the residual gradient is predominantly a flow-velocity phenomenon (Bernoulli effect) caused by blood exiting the massively dilated 40-mm proximal arch into a physiologically normal-sized 18-mm conduit, alongside mild graft angulation and persistent collateral steal. Importantly, as previously reported that adult patients undergoing extra-anatomic bypass for isolated arch obstructions often experience persistent hypertension due to chronically altered vascular compliance, underscoring the need for lifelong surveillance and medical management [2].

### Limitations

This case report has several limitations. First, the lack of longitudinal imaging or prior clinical records precludes definitive proof of the “progression” hypothesis from CoA to acquired IAA. Second, the follow-up period is short (4 months); expectations regarding the long-term durability of the graft and LV reverse remodeling remain to be prove.

## Conclusion

Severe coarctation of the aorta can present in adulthood as an acquired atresia compatible with an interrupted aortic arch. In such adult presentations, comprehensive preoperative 3D imaging is indispensable for delineating extensive collateral networks and mitigating surgical bleeding risks. In heavily collateralized adult patients, off-pump extra-anatomic bypass via thoracotomy is a safe, effective, and less invasive strategy expected to alleviate refractory hypertension and prevent devastating long-term cerebrovascular complications.

## Data Availability

The datasets used and/or analyzed during the current study are available from the corresponding author on reasonable request.

## Abbreviations

IAA: Interrupted aortic arch
CoA: Coarctation of the aorta
BP: Blood pressure
CTA: Computed tomography angiography
CT: Computed tomography
MPR: Multiplanar reconstruction

## References

[1] Salciccioli KB, Zachariah JP. Coarctation of the Aorta: Modern Paradigms Across the Lifespan. Hypertension. 2023;80(10):1970–9. 10.1161/HYPERTENSIONAHA.123.19454 PMC10530495 37476999

[2] Jiang Y, Wang C, Jiang X, Chen S. Is surgery necessary for adults with isolated interrupted aortic arch? Case series with literature review. J Card Surg. 2021;36(7):2467–75. 10.1111/jocs.15525 33844339

[3] Patel DM, Maldjian PD, Lovoulos C. Interrupted aortic arch with post-interruption aneurysm and bicuspid aortic valve in an adult: a case report and literature review. Radiol case Rep. 2015;10(3):5–8. 10.1016/j.radcr.2015.06.001 PMC4633978 26649108

[4] Messner G, Reul GJ, Flamm SD, Gregoric ID, Opfermann UT. Interrupted aortic arch in an adult single-stage extra-anatomic repair. Tex Heart Inst J. 2002;29(2):118–21. PMC116738 12075868

[5] Warnes CA, Williams RG, Bashore TM, et al. ACC/AHA 2008 Guidelines for the Management of Adults With Congenital Heart Disease. Circulation. 2008;118(23):e714–833. 10.1161/CIRCULATIONAHA.108.190690 18997169

[6] Sai Krishna C, Bhan A, Sharma S, Kiran U, Venugopal P. Interruption of aortic arch in adults: surgical experience with extra-anatomic bypass. Tex Heart Inst J. 2005;32(2):147–50. PMC1163459 16107103

[7] Garcia C, Rey J, Lopez A, Lamelas J, Orozco-Sevilla V. Left subclavian to descending aorta bypass: A less invasive extra-anatomical solution for a type A interrupted aortic arch. J Card Surg. 2019;34(10):1117–9. 10.1111/jocs.14173 31344271

